# Three-dimensional architecture of the palmar plate of the thumb metacarpophalangeal joint in infant macaque

**DOI:** 10.1007/s00795-025-00423-5

**Published:** 2025-02-08

**Authors:** Hiroko Sato, Tatsuo Shimada, Tsuguaki Hosoyama, Yutaro Shibuta, Nobuhiro Kaku

**Affiliations:** Department of Orthopedic Surgery, Faculty of Medicine, Oita University, 1-1 Idaigaoka Hazama-Machi, Yufu City, Oita 879-5593 Japan

**Keywords:** Palmar plate, Thumb metacarpophalangeal joint, Scanning electron microscopy, Infant microanatomy, Macaque

## Abstract

The palmar plate is a crucial structural part of hand, associated with metacarpophalangeal and interphalangeal joints. Pediatric disorders involving the palmar plate of thumb metacarpophalangeal joint include trigger thumb, hyperextension, instability, and dislocation. While anatomical differences exist between children and adults, detailed microstructure evaluations in infants remain unexplored. In this study, we provide a histological and structural assessment of the previously unresolved microstructure of the palmar plate in the thumb metacarpophalangeal joint of infant Japanese macaques (*Cercopithecidae*, *Macaca fuscata*), a relevant model for human development. Histological staining (light microscopy) and scanning electron microscopy were employed to visualize the three-dimensional microstructure. The palmar plate of the infant macaque was found to contain (1) elastic fibers, (2) hyaline cartilage composed of type II collagen, and (3) type I collagen fibers arranged in distinct patterns. The cartilaginous region exhibited a reticulate fiber arrangement on its periphery, while the membranous region displayed dense and complex fibers on the proximal phalanx side and parallel on the metacarpal side, respectively. This is the first comprehensive three-dimensional investigation of the infant’s thumb’s palmar microanatomy, providing a foundation for understanding its development and implications for pediatric disorders.

## Introduction

The palmar plate (PP) is a structural component located on the palmar side of the metacarpophalangeal joint (MPJ) and interphalangeal joint (IPJ). It comprised a membranous proximal part, which becomes tensed during extension and folds during flexion, and cartilaginous distal part. In the MPJ, the PP contributes to the formation of the phalangeal rotator cuff, while in the IPJ, it forms the three-dimensional (3D) ligament box complex (volar ligament complex), [1, 2] which provides stability to the joint.

In the adult thumb, the PP exhibits unique anatomical features, including sesamoid bones that distinguish it from the other digits. As the opposable digit, the thumb plays a critical role in hand function, underscoring the importance of elucidating its microanatomy.

Pediatric disorders involving the PP of the thumb MPJ include trigger thumb [3–6], hyperextension or instability [4, 6–9], and dislocation [10–12], as reported in case studies. In addition, even mild thumb MPJ hyperextension (instability), though often not requiring treatment, can negatively impact daily activities and should not be overlooked. Hyperextension of the thumb MPJ may reduce grip strength [8], raising concerns about potential declines in quality of life during the critical stages of rapid growth and development in infancy. Given the prevalence of thumb-related disorders in infancy, detailed investigation of the microanatomy is essential for understanding these conditions. However, the anatomy of the infant thumb, notably lacking sesamoid bones, remains insufficiently studied.

Anatomical differences exist between the PP of the MPJ (MPJPP) in children and adults. In the thumb, sesamoid bones begin to ossify around the age of 10 and are absent in infants [13–15]. These sesamoids provide the insertion sites for the abductor pollicis brevis, adductor pollicis, and flexor pollicis brevis muscles, functioning as dynamic stabilizer of the thumb MPJ [16]. Therefore, thumb MPJPP disorders in infants may be associated with underdevelopment of the sesamoid bones [4–6, 10–12].

Previous studies have explored the microanatomy of the MPJPP in adults. Tan et al. [16] conducted a histological evaluation of the thumb MPJ in human adults and reported that the PP comprises a single layer of hyaline cartilage surrounding the sesamoid bones. This contrasts with the findings in other adult human fingers, where the MPJPP is fibrocartilage and consists of three distinct layers of fibers [1, 2, 17–20]. The microstructure of the thumb MPJPP in infants likely differs from that in adults because of the absence of developed sesamoid bones, highlighting the need for further investigation. Moreover, purely histological assessments, such as immunohistochemical staining, are insufficient for understanding the 3D microanatomical structure. However, ethical and societal constraints on obtaining pediatric human specimens have limited histological and electron microscopic evaluations of the microstructure of the infant thumb MPJPP.

In this present study, we aimed to address this gap by providing a detailed histological and structural analysis of the previously unreported microstructure of the human infant MPJPP. Specimens were obtained from the same strain of infant Japanese macaques (family *Cercopithecidae*) [21–23], offering valuable insights into the developmental anatomy of the thumb MPJPP.

## Materials and methods

### Sample preparation

The right hand thumb of a Japanese infant macaque (*Macaca fuscata*, Toyo, Tokyo, Japan) fixed in 10% formalin was sectioned along the median plane through the thumb MPJ region, including the distal, proximal, and metacarpal phalanges.

The study was conducted ethically in accordance with the Declaration of Helsinki and was approved by the Ethics Committee of Oita University (approval number, A036003; approval date, 1 April 2014).

### Light microscopy (LM)

The MPJ region of the thumb was fixed either in 10% formalin or 4% paraformaldehyde in phosphate-buffered saline (pH 7.4), decalcified in 4% ethylenediaminetetraacetic acid solution for 3 days, and sectioned along the median plane. Subsequently, the samples were dehydrated in alcohol and embedded in paraffin. Serial sections (7 µm thick) were prepared and stained with hematoxylin and eosin (H and E) or dual-stained with aldehyde fuchsin and light green (AF and LG) and alcian blue (AB). Stained specimens were observed under a light microscope (Nikon, Tokyo, Japan; Keyence, Osaka, Japan).

### Scanning electron microscopy (SEM)

To visualize the collagen fibers using SEM, paraffin blocks previously prepared for LM were deparaffinized with xylene and rehydrated. The samples were then refixed in Karnovsky’s fixative (2.5% glutaraldehyde and 2.0% paraformaldehyde) and immersed in 2 N sodium hydroxide at 37 °C for 3 h to digest the extracellular matrix [24], thereby exposing the collagen fibers. The samples were subsequently stained for conductivity by treating them sequentially with 1% osmium tetroxide and 1% tannic acid, and a second treatment with 1% osmium tetroxide (1 h each). The samples were then dehydrated by gradually increasing the concentration of ethanol, freeze-dried using *tert*-butanol, and gold-coated. The prepared specimens were examined using a Hitachi S-800 scanning electron microscope (Hitachi, Tokyo, Japan) at an accelerating voltage of 5 kV. The diameters of the collagen fibers were measured using Java ImageJ software (version 1.46d, Wayne Rasband, National Institutes of Health).

### 3D images

The LM and SEM findings were integrated using Adobe Photoshop (2024). SEM images were colored to enhance visualization of the 3D microstructure of the MPJPP of the thumb.

## Results

### LM findings

**Fig. 1:**
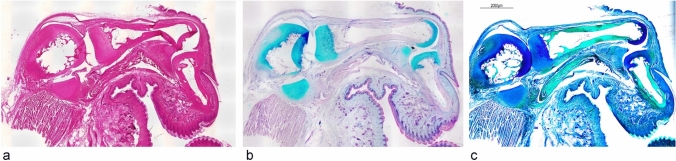
Low-magnification light micrograph of the sagittal section of the thumb in an infant macaque. **a** Hematoxylin and eosin (H and E) stain. **b** Alcian blue (AB) stain: the cartilage of the distal, proximal, and metacarpal phalanges, as well as the palmar plate (PP), are AB-positive. **c** aldehyde fuchsin (AF) and light green (LG) stain: the fibrous area of the cartilaginous part is made up of a mixture of collagenous and elastic fibers

The PP consists of cartilaginous and membranous components, with the membranous part surrounding the cartilaginous portion (Fig. 1a). Furthermore, both the articular cartilage and the cartilaginous part of the PP stained positively for AB at pH 2.5, indicating the presence of acidic sugars such as hyaluronic acid (Fig. 1b). In dual staining with AF and LG, bone tissue and tendons appeared green, whereas the membranous component of the PP displayed green collagen and purple elastic fibers (Fig. 1c).

**Fig. 2:**
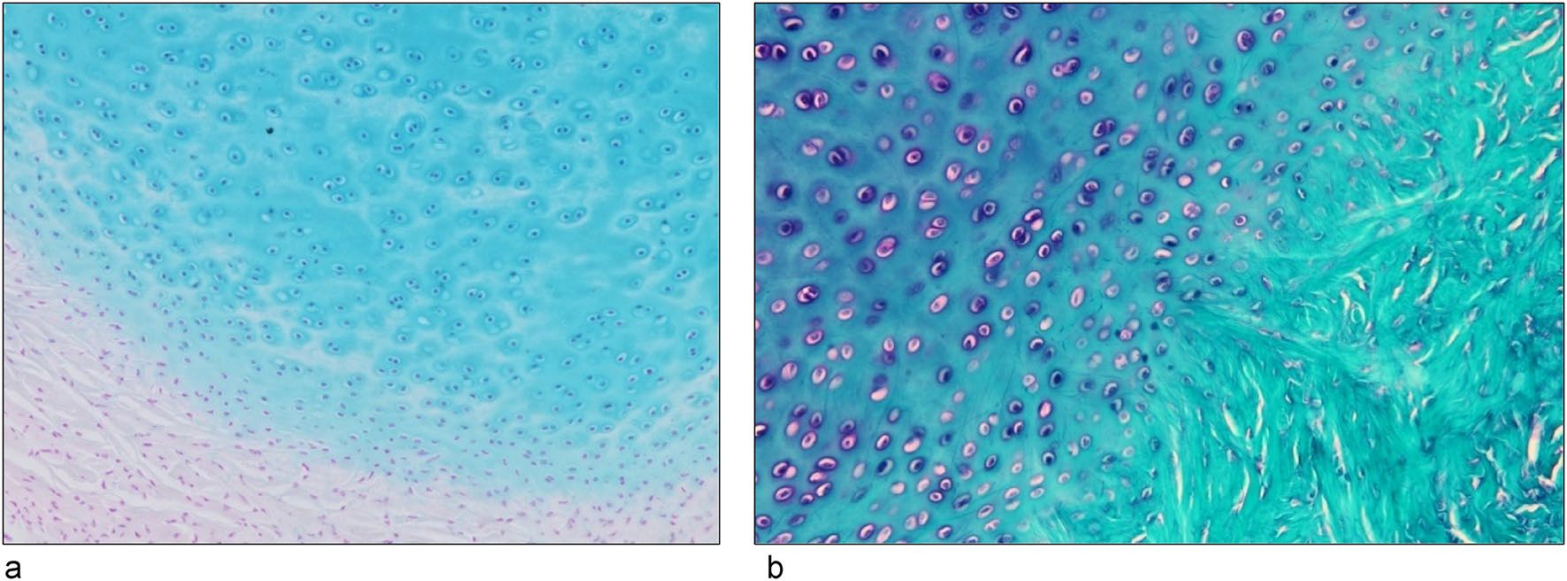
Light micrograph of the cartilaginous part of the palmar plate (PP) (magnification,  × 200). **a** Cartilaginous part is dominated by hyaline cartilage and its substrate proteoglycans. Fibroblasts are observed in the perichondral region. **b** Fibrous area around the cartilaginous part is composed of collagen fibers stained green with light green (LG) stain and elastic fibers are stained purple with aldehyde fuchsin (AF) stain

The PP of the MPJ was also thoroughly examined. The cartilaginous part of the PP in the MPJ was morphologically distinct, with small chondrocytes at the margins and two to three groups of large chondrocytes centrally located. The periphery of the central chondrocytes showed strong positivity, confirming the presence of hyaline cartilage, similar to articular cartilages (Fig. 2a). However, the fibrous region at the periphery of the cartilaginous area consisted of dense connective tissue and numerous fibroblasts (Fig. 2a), predominantly collagenous with elastic fibers (Fig. 2b).

**Fig. 3:**
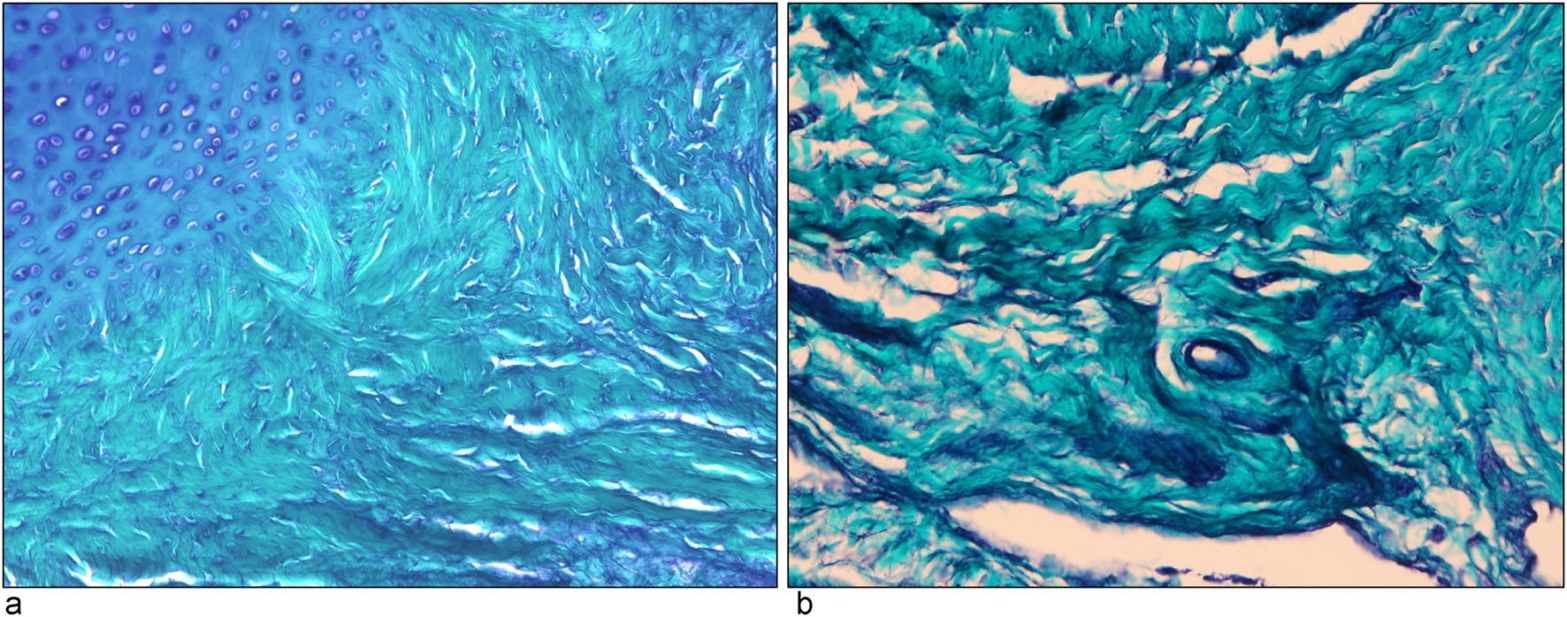
Light micrograph of the membranous part in aldehyde fuchsin (AF) and light green (LG)-stained palmar plate (PP). **a** Proximal phalanx side (magnification,  × 200). **b** Metacarpal side (magnification,  × 400). Elastic fibers (purple) are present in **a** and **b**, and are particularly dense in **a**

The membranous part of the PP showed abundant collagenous and elastic fibers (Fig. 3a and b). It encased the cartilaginous part, with the proximal phalanx side of the membranous part showing short, thick, and complexly arranged collagen fibers (Fig. 3a). In contrast, the metacarpal side of the membranous part was elongated and cord-like, composed of dense, parallel collagen fibers (Fig. 3b).

### SEM findings

**Fig. 4:**
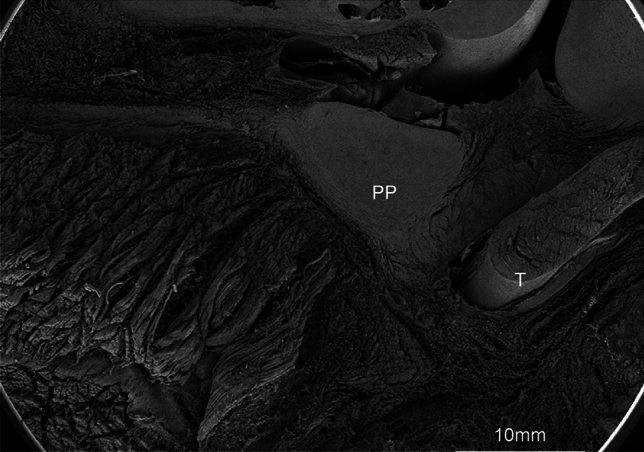
Scanning electron microscope image of sagittal section of the thumb in the infant macaque (magnification,  × 30). Scale bar = 10 mm. (*PP* palmar plate, *T* flexor pollicis longus tendon). The PP comprises a thick cartilaginous part and an elongated membranous part

SEM findings revealed that the proteoglycans were the only components degraded, revealing the 3D structure of the cartilage tissue and tendons. The PP’s membranous part on the proximal phalanx side was notably thick (6 µm), short, densely complex, and attached to the flexor pollicis longus tendon. In contrast, the metacarpal side of the membranous part was long and slender (length; 20 µm) and consisted of parallel bundles of collagenous fibers (Fig. 4).

**Fig. 5:**
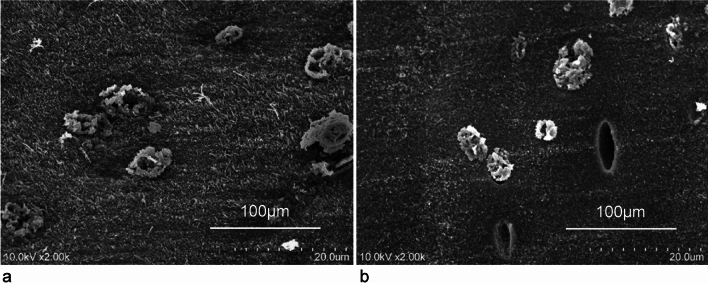
Scanning electron microscope image of the cartilaginous part of the metacarpophalangeal joint (MPJ). **a** Palmar plate (PP) cartilage. Similar to articular cartilage **b**, it is composed of chondrocytes and a matrix; however, the chondrocytes are slightly larger. **b** Articular cartilage. Cartilage lumen visible. Scale bar = 10 µm

Chondrocytes in MPJPP cartilage were approximately 100 µm in diameter and organized into approximately 2–3 groups (Fig. 5a).

**Fig. 6:**
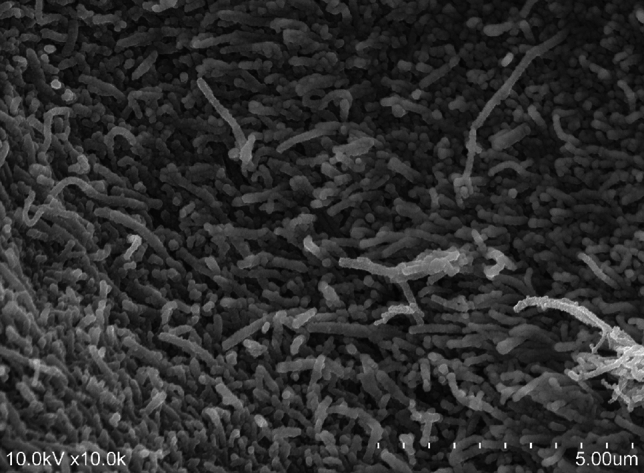
Enlarged scanning electron microscope image of the substrate of the cartilaginous part. Type II collagen fibers with approximately 80 nm diameter are densely packed

The cartilaginous substratum was densely packed with type II collagen fibers measuring approximately 80 nm in diameter (Fig. 6).

**Fig. 7:**
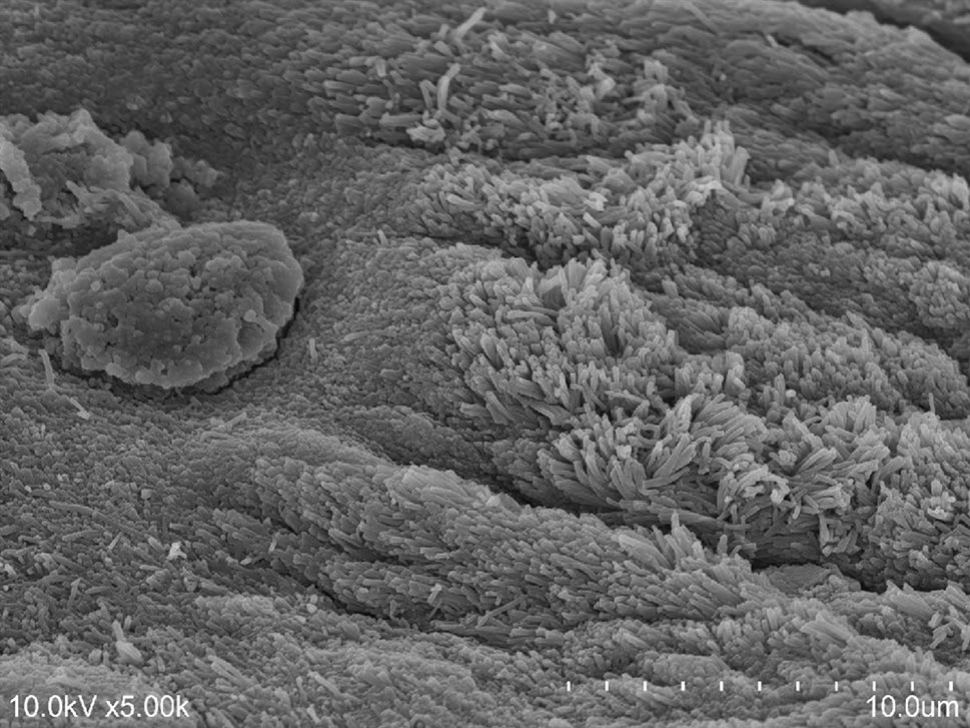
Scanning electron microscope image of the boundary between the cartilaginous and the fibrous parts. Some of the collagen fibers in the fibrous area had plunged into the cartilaginous part

The boundary between the cartilaginous and fibrous regions consisted of type I collagen fibers, each measuring approximately 120 nm in diameter, arranged in a reticular pattern, with some fibers extending into the cartilaginous part (Fig. 7).

**Fig. 8:**
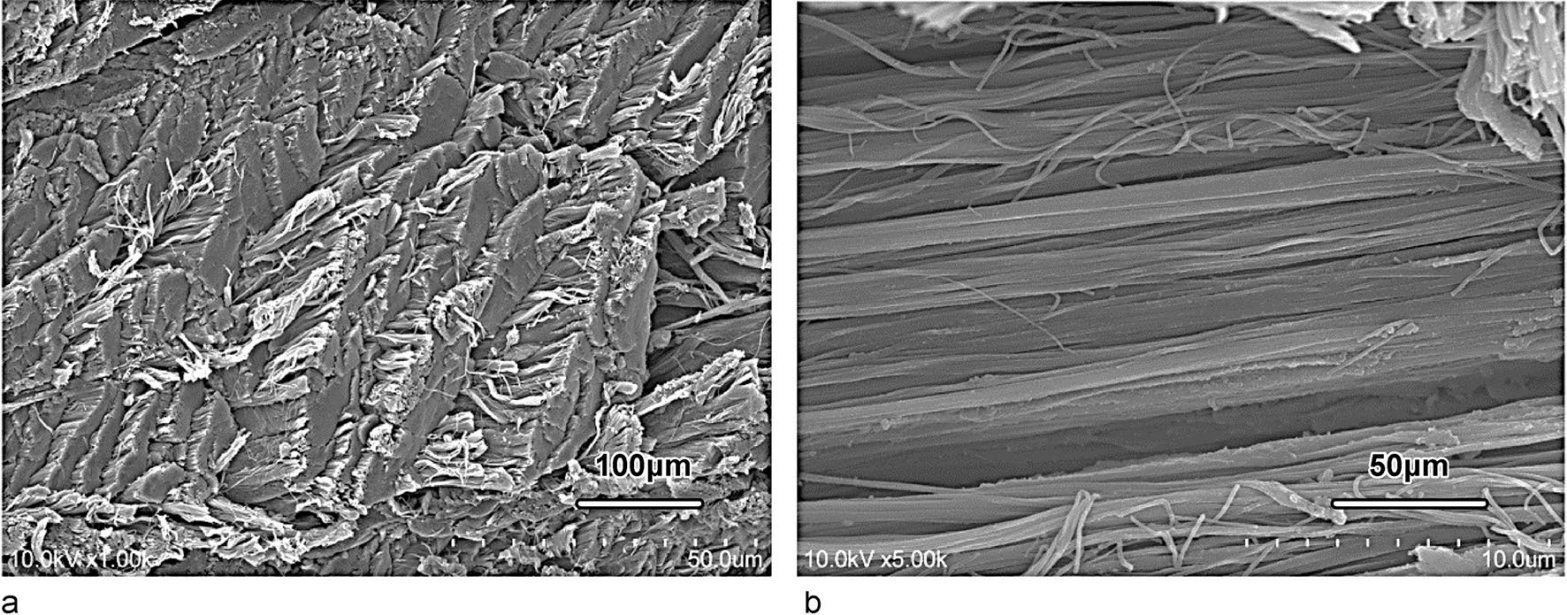
Scanning electron microscope image of the flexor pollicis longus tendon. The flexor pollicis longus tendon consisted of dense connective tissue organized in parallel bundles of collagen fibers, each with an approximate diameter of 120 nm. Scale bar = 100 µm (**a**) and 50 µm (**b**)

The flexor pollicis longus tendon was attached to the proximal phalanx side of the PP (Fig. 8).

**Fig. 9:**
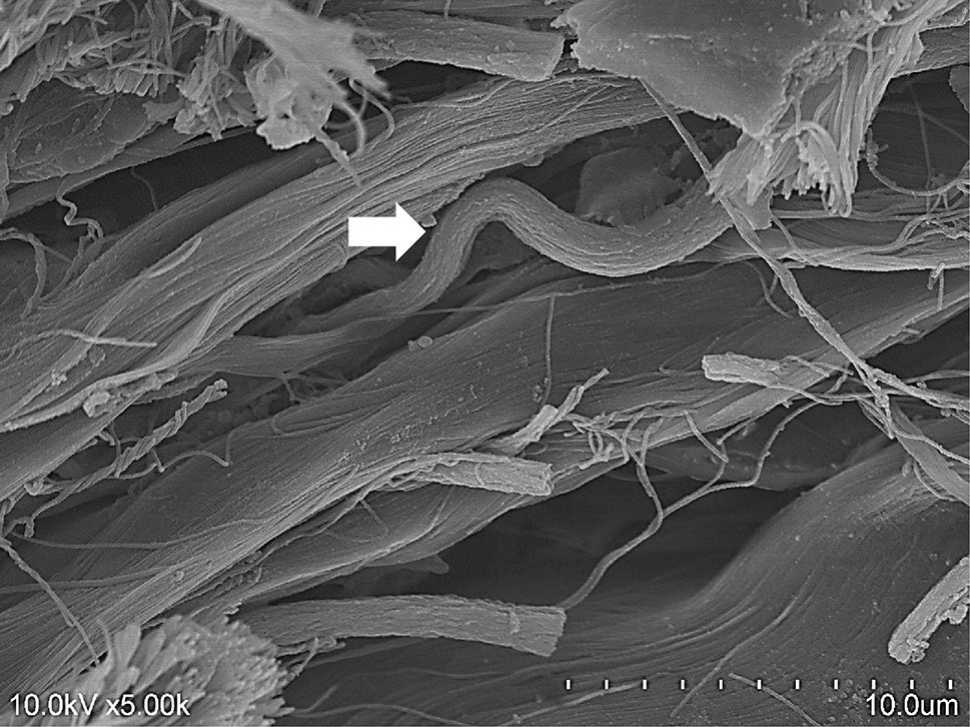
Scanning electron microscope image of the membranous part. Tortuous elastic fibers (arrow) ran between the fiber bundles

In the membranous part of the PP, tortuous elastic fibers were observed to be interspersed between the parallel-aligned collagen fibers bundles (Fig. 9).

**Fig. 10:**
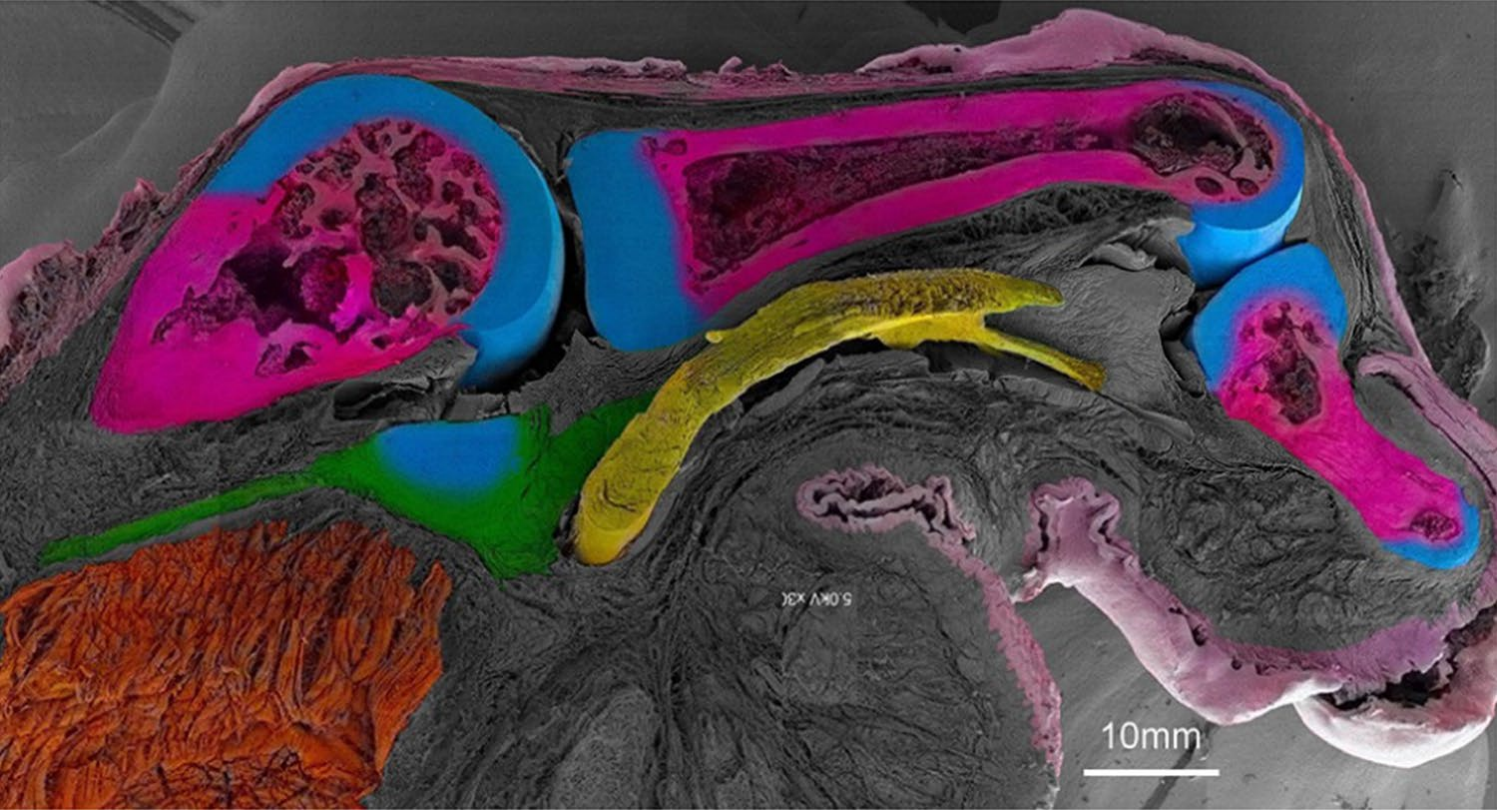
Three-dimensional images of the MPJPP of the thumb of an infant macaque with histological evaluation (magnification,  × 30). Skin/pale pink; bone/dark pink; cartilage/light blue; membranous part/green; flexor pollicis longus tendon/yellow; muscle/orange

Notably, in this study, we combined 35 photographs captured at a very low magnification (30x) into a single image using Adobe Photoshop (Fig. 10). This allowed for the correlation of the 3D images with histological evaluations, revealing the 3D microstructure and location of the MPJPP of the thumb in infant macaques (Fig. 10). The MPJPP consists of a cartilaginous part and an elongated membranous component. The membranous component, attached to the cartilaginous part, is shorter on the proximal phalanx side and elongated on the metacarpal side. The metacarpal side of the membranous part exhibited parallel dense connective tissue, similar to the flexor pollicis longus tendon, with a notable abundance of elastic fibers.

## Discussion

We conducted a detailed histological and structural evaluation of the microstructure of the MPJPP in infant macaques utilizing specimens from the same lineage of Japanese macaques (*Cercopithecidae*). We employed histological staining techniques, including H and E, AF and LG, and AB, to identify and compare tissue structures. In addition, SEM was used to further explore the 3D arrangement of collagen and elastic fibers. Finally, we correlated and color-coded the findings from the LM with those from the SEM to visualize the 3D microstructure of the PP of the thumb.

The presence of elastic fibers was confirmed in 3D SEM images (Fig. 9). Elastic fibers, primarily composed of elastin, are known for their rubber-like or spring-like elastic properties, which contribute to the resilience and flexibility of various tissues. These fibers are abundant in organs such as the arteries, lungs, and subcutaneous dermis, where their elasticity plays a critical functional role. In joints, elastic fibers are predominantly found in the synovial membranes of the joint capsule [25], intervertebral discs [26], and ligamentum flavum [27], where they stabilize and restore joint motion and protect against mechanical impacts. The MPJ lacks a checkrein ligament, which makes it unable to adequately restrain hyperextension [28]. Based on our findings, we hypothesized that elastic fibers observed the membranous part partially infiltrate the fibrous cartilaginous part, which, together with the strength provided by the collagen fibers, serves to resist hyperextension.

The cartilaginous part of the PP is reportedly the fibrocartilaginous tissue [1, 2, 17–20]. However, the articular labrum, which represents fibrocartilage, contains type I collagen, which is not detected in the cartilaginous part of the MPJPP of the thumb in our study [29, 30]. This distinction aligns with previous observations by Tan et al. [16], who suggested the presence of hyaline cartilage around the sesamoid bones of the MPJPP in the adult human thumb. However, their conclusions were derived solely from only staining methods, leaving room for further clarification [16]. In contrast, we observed that chondrocytes were present in clusters of two or three, and the cartilage matrix was filled with collagen fibers (type II collagen) with an approximate diameter of 80 nm, and exhibited a random orientation (Figs. 5a and 6). These findings indicate that the cartilaginous part of the MPJPP likely serves lubricative and protective functions similar to articular cartilage.

Furthermore, it was confirmed that the fibrous area of the PP predominantly contained collagen fibers composed of type I collagen (Fig. 7). This finding aligns with previous study by Ché et al. [31], who demonstrated that the PP primarily comprises type I collagen with a small amount of type III collagen. Type I collagen provides tensile strength to tendons and fascia, whereas type III collagen is widely distributed in tissues that contain type I collagen, contributes to mixed collagen fibers [32]. Therefore, to elucidate whether the PP is more analogous to ligament, tendon, or fascia, further research into the detailed molecular and ultrastructural properties of the collagen component is required [1, 2, 17–20].

The collagen fibers on the proximal phalangeal side of the thumb MPJPP of the infant macaque were observed running perpendicular to the long axis of the thumb (Fig. 4), which was consistent with the report by Tan et al. in adult human [16]. In addition, Tan et al. [16] reported that the proximal part of the palmar plate was composed of loose connective tissue; however, our investigation revealed that dense connective tissue was the main component in this region (Fig. 3b). The specimen examined by Tan et al. was a coronal section, while we used sagittal sections. In our sagittal sections, the loose connective tissue observed on the articular side was determined to be most likely the joint capsule (Figs. 1c and 4). This discrepancy may be related to differences in the direction of sectioning, variations in age, or species, and requires further investigation.

The movement of the condylar joint (MPJ) is characterized by its multidirectional structure. In addition, the MPJ lacks a checkrein ligament and thus, cannot suppress hyperextension. However, the proximal phalanx side of the PP can withstand loads during hyperextension owing to its dense reticular arrangement and thick collagen fibers firmly attached to the bone. In contrast, the metacarpal side is composed of abundant elastic fibers and dense, parallel collagen fibers that can withstand repeated longitudinal movements of flexion and extension.

Hyaline cartilage in the cartilaginous part may be associated with the formation of the sesamoid bone in the MPJPP of the thumb. This association arises from the possibility that hyaline cartilage represents a cartilaginous nodule of the sesamoid primordium [13, 14, 33]. Therefore, the infant MPJPP may be inherently unstable due to the incomplete stabilization provided by the sesamoid bones [16]. Thus, hyperextension or instability associated with infant thumb MPJ may be considered for active treatment and monitoring of the progress of sesamoid bone development [5].

This study has some limitations. First, we used the Japanese macaques as the study model. While monkeys are among the closest relatives to humans, their MPJPP is not necessarily equivalent to the human MPJPP. This is because the MPJPP of the human thumb, who are upright, bipedal, and fully released, may differ from that of other primates. On the other hand, primate hands adapted from quadrupedal forelimbs for arboreal grasping, may still retain common structural and functional characteristics. Second, the study cohort was small. The size of the cohort was reduced owing to the social and ethical contexts, which made it difficult to obtain monkey and human infant samples annually. Last, the analysis was confined to sagittal plane sections.

## Conclusion

The significance of this study is that the fine structure of MPJPP in human infants, which has not been previously studied, was histologically and structurally examined using specimens from infant Japanese macaques (*Cercopithecidae*) of the same lineage. We histologically and structurally evaluated the fine structure of the MPJPP of the thumbs of infants before sesamoid bone formation. The results confirmed the presence of elastic fibers, with the cartilaginous part identified as hyaline cartilage composed of type II collagen. The structural features of the collagen fibers varied: they formed reticulate pattern on the periphery of the cartilaginous part, were dense and complex on the proximal phalanx side, and parallel on the metacarpal side of the membranous part. These findings are expected to be useful in elucidating the pathology and treatment of diseases associated with the MPJPP of the thumb in children, provided further high-quality research is conducted.

## Data Availability

The data generated in the present study are included in the figures of this article.
